# Institutional Outdoor Work and Administration

**Published:** 1911-08-26

**Authors:** 


### SPECIAL INSTITUTIONAL ARTICLE.

### INSTITUTIONAL OUTDOOR WORK AND ADMINISTRATION.

By A SANATORIUM SUPERINTENDENT.

[The Editor will be pleased to receive criticisms and suggestions of the application of the principle underlying this article. So many institutions have their own methods for producing various needs that an interchange of experience would be of the greatest value to the whole institutional world.]

### I.—INTRODUCTORY.

One of our judges lately gave it as his opinion that prisons should be self-supporting to a much greater extent than at present, as regards both commissariat and such items as repairs and renewals. There are obvious advantages in such a plan, advantages to the institution as well as to its inmates, be that institution penal or of any other character. The latter half of the nineteenth century saw this excellent practice coming into vogue at mental hospitals, which at the present time probably do most in this direction—at any rate, more than other medical institutions.

#### Why Outdoor Work is Popular.

The reasons for this are their secure financial position, State maintained and directed, and their great size and large number of inmates, who are certainly mentally but not often physically disabled. The long period of time necessary for the modern treatment of consumption, and the importance of securing suitable employment after this for arrested cases of the disease, have inspired the establishment or the wish for farm colonies in connection with sanatoriums, where convalescent patients may be maintained, if not without cost, at any rate very cheaply, until the notorious risk of relapse is averted. The plan of every lately-started sanatorium contains some provision for such development; and since the proposed National Insurance Bill, whatever happens to modify it is, at any rate, likely to increase the number of these institutions, and the scope of already existing ones, it is here proposed to set down such elementary principles of farm and estate work as are relevant.

#### Outdoor Work in Sanatoriums.

Sanatoriums are the institutions to which this short series of articles will especially apply, but medical or lay officers of other institutions may also find points of interest therein. The word interest is used advisedly. An ill-natured Frenchman has remarked, *apropos* of the general taste for bright colours and brass bands, that there is a nigger in everyone of us. Be that as it may, there are few men but have somewhere in them a touch of the farmer. True, the capability may not be as widely distributed as the taste. Agriculture and stock-raising in their rudiments are not, however, highly technical matters, as witness the success of small-holders. And not much more than these rudiments are all that can be attempted by small institutions, unable as they are to afford the steward or farm bailiff found at asylums. Nowadays, too, one can find useful handbooks on every subject under the sun. The real essential is elbow-grease, which will always in the long run beat expert knowledge, which is not wholly disinterested and conscientious. To secure this essential, the person at the head of affairs must have marked keenness for the work, such as will lead him to look into every detail.

#### The Increasing Purpose.

Even if great keenness be not present to begin with, some considerable appetite will come in eating. Was it not Bacon who remarked that a man had much better buy an undeveloped than a complete estate, on account of the pleasure of laying it out? The "increasing purpose," the delight in progress continually accelerating, the pleasure of having a free hand and one's own way in arranging things, contain a deep attraction for human nature. If only some keenness and conscientiousness coincide with even moderate ability, then the financial result—what one's committee look at—will certainly be favourable.

#### Thoroughness in Administration.

The endeavour to develop all possible resources will lead to such a thorough looking into all administrative departments as cannot fail to promote economy. To take a small and homely instance: the head of an institution who wants to make something of the pigs will look sharply after the potato peelings; probably he or she will then find that the refuse tubs contain much that ought not to be there—even scarcely-touched joints of meat, perhaps, for institutional domestics, for some occult reason, are peculiarly and wickedly wasteful. Or the matron's desire to do well with the poultry will lead her to invest thirty shillings in a bone-crusher and thus convert an almost waste product into those necessary articles, eggs, which seem to be growing dearer every year.

#### Eliminating the Middleman.

The consumer who sets out to supply himself is in a favourable position to begin with, for he is sure of his market and can save all middleman profits. If he begin thus on a sure foundation, and in a small way, with no expenditure on showy unessentials, and works hard, he will surely do well. The motto, in North Country phrase, must be: "Not bonny, but *good.*" In ensuing articles it is proposed to treat shortly of potato-growing, pig-keeping, and the large subject of poultry, with a final budget of general hints on such development of grounds and land as comes within the sphere of the institutional officer.

### II.—POTATO-GROWING.

At first sight it may seem superfluous to enlarge upon such a simple operation of husbandry as the growing of potatoes, since somewhere about Good Friday nearly every cottager may be seen planting his rows. But then the cottager, or the ordinary groom-gardener, has no notion of the advantage of cultivating early varieties, or of obtaining good Scotch seed, or of the other crops that can be worked in with potatoes. And he sticks year after year to names that have long outlived their reputations. Or again it may be objected that it is practically as cheap to buy as to grow. This last point must depend a little upon local circumstances; but as an institution with a daily average of only fifty to sixty head to cater for will require fully thirty stone of potatoes a week, or about ten tons a year, and as at the ordinary sanatorium the necessary land will be rent free, and a fairly constant supply of labour also gratuitous, one can hardly expect to buy from the grocer as cheaply as the cost of home-growing under the above conditions. Ten tons of potatoes at six shillings for ten stone comes to about £45, and as a matter of fact, even using the best seed from nurserymen, the year's home-grown crop will cost nothing like this. Besides, the small tubers—those too small for the table—make useful food for stock, as also do the tops of early potatoes.

### Value of Potatoes as First Crop.

Again, to bring pasture or moorland into cultivation, potatoes are one of the best first crops. They will even succeed the first year without manure (although the plan is not altogether advisable), since nitrogen accumulates in the top layer of old pasture, and in their rapid growth they kill all the weeds and thistles, leaving the land clean and ready for other crops. Then, as mentioned, it is possible to get with certain kinds a quickly succeeding crop of Scotch cabbage and kale, which will provide greenstuff in the winter for cows and pigs, or even for the table. Thus an acre or so of poorish land may be made to pay abundantly for the labour and manure expended upon it.

### How to Reduce the Fencing Bill.

Sanatoriums are generally found located upon poorish land, and as the institutional garden will probably be reserved for better things than potatoes, it may be necessary to enclose a piece. Here one comes at once upon the question of fencing, and again it may well be feasible to economise by home management. Sanatoriums nearly always have a plantation in the grounds, and most plantations contain at any rate a few larches and oaks. Just as commonly, too, woods are rather under than over-thinned. Nearly every countryside boasts a sawmill. By cutting the trees into 4 feet 6 inch lengths, and by sending the thick portions to be sawn into 3 inch square posts, a saving of about sixty per cent. will be effected. The trees should be felled and allowed to lie all winter, if possible, since the frost will drive out the sap and hasten seasoning. If possible, too, the posts should be stacked for a while before use, first out of doors and then under cover. But good oak or larch has naturally a very long life in the ground. The boles of medium sized trees must be cut longer and kept for corner posts to take the strain of the wire; this and staples can be got cheaply, while most farmers own, and, in the pleasant country fashion, will lend, a wire tightener. With the supervision of a handy man, a small plot of land can thus be easily fenced in. If rabbits or lambs abound, it will be well to apply in addition three-foot rolls of two-inch mesh-wire netting, sinking them a foot into the ground. Deeper than this, providentially, a rabbit has not got the sense to countermine.

### The Times for Planting.

The aim should be to get potatoes in the best condition all the year round, and particularly as early as possible in the summer, for new potatoes are the dearest to buy. Much can be done by sprouting them in boxes, and carefully planting at the earliest opportunity. With these early potatoes there is, however, the risk of late frosts to reckon with, and the young plants should be covered with branches if frost threatens. The preparation of the land, if broken up for the first time, should be begun in autumn with a good ploughing up. By the early spring the frost will have brought the surface to a good tilth. If there is plenty of labour available the rows can be dug by hand, laid with manure, and filled in after marking each end with a stake. A line is then put between the two stakes and the seed potatoes (which need no cutting) dibbled in along it at intervals of a foot. The rows should be two feet apart. The other way is to hire a farmer with a drill plough, which is much quicker. Early, second early, and main crop varieties, Scotch-grown, are thus set, the last being for winter use. It should be a good keeper; if possible, like the Royal Kidney, with a small top. The early kinds will soon be ready for ridging up, and the others in turn. In the furrows now opened, and after a spell of rainy weather, Scotch cabbage and curly-headed kale, which have been struck from seed in the garden, should be dibbled in, to "come away" well after the potatoes are lifted.

### The Storing.

In this manner a supply will continuously come ready for use, obviating the necessity of storing all at once. Some storage room will, however, be needed, and it is not generally known that on a concrete floor perfectly ripened potatoes will keep quite well in the open air. Pits may be used, but in storing potatoes as in keeping them for seed, the great point is maturity. In the next article the subject treated of will be the pork and bacon supply of the institution; and as already seen the above directions provide for a good deal of the necessary pig-food.

(*To be continued.*)

